# P-1706. Understanding Facility Bed Size Changes in NHSN over the COVID-19 Public Health Emergency

**DOI:** 10.1093/ofid/ofae631.1872

**Published:** 2025-01-29

**Authors:** Olivia Arar, Lisa Iguchi, Elizabeth Breitenstein, Alexia Y Zhang, Gregory B Tallman, Dat Tran, Jessina C McGregor

**Affiliations:** Oregon State University, Portland, Oregon; Oregon Health Authority, Portland, Oregon; Oregon Health Authority, Portland, Oregon; Oregon Health Authority, Portland, Oregon; Providence Health & Services Oregon, Portland, Oregon; Oregon Health Authority, Portland, Oregon; Oregon State University, Portland, Oregon

## Abstract

**Background:**

Antimicrobial use surveillance data within National Healthcare Safety Network (NHSN) uses patient care location mapping data to define locations and inform risk adjustment. During the COVID-19 public health emergency, many hospitals changed how beds were utilized. This study compares location mapping changes over time within a statewide hospital antimicrobial stewardship collaborative during the COVID-19 public health emergency.

**Figure 1.**
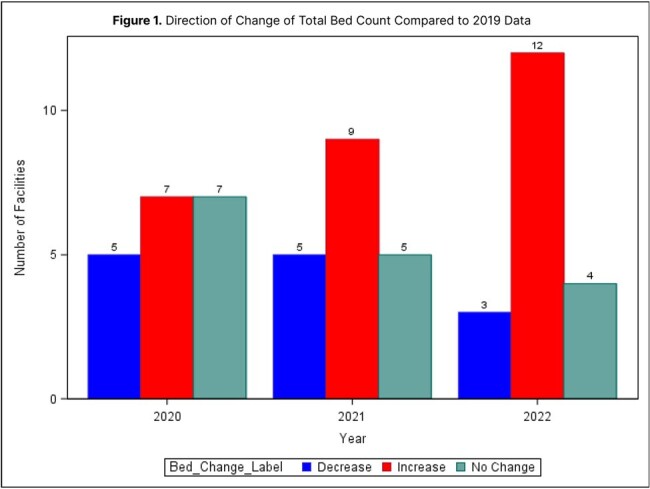
Frequency of Facilities by Change in Total Beds from 2019

In this analysis, each facility's change in total bed count was subtracted from the 2019 value to classify the change as an increase, decrease, or no change in each year of analysis. The figure demonstrates that total bed counts varied in the direction of change; there was no significant difference in the direction of change across years (p=0.63) .

**Methods:**

We utilized facility survey data from facilities who submitted data from 2019-2022 to the Oregon Antimicrobial Stewardship Network (ORASN) NHSN group. Annual total bed and ICU bed counts were compared to 2019 data for each facility. Changes in bed counts were also categorized as increases, decreases, and no change and compared using the Fisher’s exact test.

**Figure 2.**
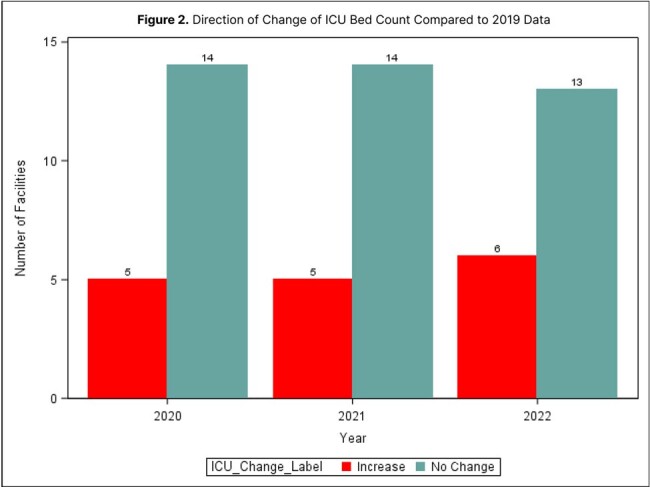
Frequency of Facilities by Change in ICU Beds from 2019

In this analysis, each facility's change in ICU bed count was subtracted from the 2019 value to classify the change as an increase, decrease, or no change in each year of analysis. The figure demonstrates that ICU bed counts increased in all years; there was no significant difference in the direction of change across years (p>0.99) .

**Results:**

Nineteen hospital facilities contributed sufficient data and were included in analysis. Overall, there was a mean decrease in total bed count in 2020 (-8.16 ± 26.68) and 2021 (-4.16 ± 29.3) but an increase in 2022 (27.89 ± 68.39) when compared to 2019 baseline total bed counts at each facility. The total bed counts decreased in 8.77% of facilities in 2020 and 8.77% in 2021; while increases were observed among 21.05% in 2022 (*p* = .63; see Figure 1). There was a mean increase in the total number of ICU beds in 2020 (3.95 ± 9.51), 2021 (4.47 ± 10.89), and 2022 (4.63 ± 10.05) when compared to 2019 baseline data. The ICU bed counts increased in 8.77% of facilities in 2020, 8.77% in 2021, and 10.53% of facilities in 2022 (*p* > .99; see Figure 2).

**Conclusion:**

The data suggest that location mapping changed meaningfully across the COVID-19 public health emergency with no clear trend. Interpretation of the antimicrobial use data over time and risk adjusted metrics must consider these shifts. Further efforts are needed to understand NHSN location mapping changes in the context of actual historical bed utilization during this time frame.

**Disclosures:**

**All Authors**: No reported disclosures

